# Regulatory Action of Plasma from Patients with Obesity and Diabetes towards Muscle Cells Differentiation and Bioenergetics Revealed by the C2C12 Cell Model and MicroRNA Analysis

**DOI:** 10.3390/biom11060769

**Published:** 2021-05-21

**Authors:** Natalya V. Khromova, Anton V. Fedorov, Yi Ma, Kirill A. Kondratov, Stanislava S. Prikhodko, Elena V. Ignatieva, Marina S. Artemyeva, Anna D. Anopova, Aleksandr E. Neimark, Anna A. Kostareva, Alina Yu. Babenko, Renata I. Dmitrieva

**Affiliations:** 1 National Almazov Medical Research Centre, Institute of Molecular Biology and Genetics, 197341 Saint-Petersburg, Russia; antonfedorow@gmail.com (A.V.F.); mayi29082@yandex.ru (Y.M.); kondratovk.kirill@yandex.ru (K.A.K.); stanislava.prikhodko@gmail.com (S.S.P.); ignateva_ev@almazovcentre.ru (E.V.I.); marinaart888@mail.ru (M.S.A.); anopova.ann@gmail.com (A.D.A.); neymark_ae@almazovcentre.ru (A.E.N.); anna.kostareva@ki.se (A.A.K.); alina_babenko@mail.ru (A.Y.B.); renata.i.dmitrieva@gmail.com (R.I.D.); 2 Center for Molecular Medicine, Department of Women’s and Children’s Health, Karolinska Institute, 17177 Stockholm, Sweden

**Keywords:** murine myoblast cell line, mitochondria, miR-378a-3p, skeletal muscle differentiation, skeletal muscle bioenergetics, plasma, obesity, diabetes

## Abstract

Obesity and type 2 diabetes mellitus (T2DM) are often combined and pathologically affect many tissues due to changes in circulating bioactive molecules. In this work, we evaluated the effect of blood plasma from obese (OB) patients or from obese patients comorbid with diabetes (OBD) on skeletal muscle function and metabolic state. We employed the mouse myoblasts C2C12 differentiation model to test the regulatory effect of plasma exposure at several levels: (1) cell morphology; (2) functional activity of mitochondria; (3) expression levels of several mitochondria regulators, i.e., *Atgl*, *Pgc1b*, and miR-378a-3p. Existing databases were used to computationally predict and analyze mir-378a-3p potential targets. We show that short-term exposure to OB or OBD patients’ plasma is sufficient to affect C2C12 properties. In fact, the expression of genes that regulate skeletal muscle differentiation and growth was downregulated in both OB- and OBD-treated cells, maximal mitochondrial respiration rate was downregulated in the OBD group, while in the OB group, a metabolic switch to glycolysis was detected. These alterations correlated with a decrease in *ATGL* and *Pgc1b* expression in the OB group and with an increase of miR-378a-3p levels in the OBD group.

## 1. Introduction

Over the past few decades, a dramatic worldwide increase in the incidence of obesity and diabetes mellitus type 2 (DM2) has been observed. More than 600 million people suffer from obesity [1], and about 400 million have T2DM [2]; these two pathologies are closely related, since one-third of obese patients suffer from T2DM, while about 80% of T2DM patients are overweight [3,4,5].

Both obesity and T2DM pathologically affect many tissues, including skeletal muscles, causing severe functional and metabolic changes. It is well documented that skeletal muscle pathological changes common in obesity include morphological and metabolic alterations, sarcopenia, a shift towards a fast-switch fiber phenotype, a decline in the contractile function [6,7,8,9,10,11,12]. Moreover, obesity can lead to the impairments in molecular mechanisms that regulate skeletal muscle satellite cells activation, which results in altered mechanisms of skeletal muscle regeneration, development, and contractile activity [13,14].

Because muscle tissue occupies a significant part of the human body, it is one of the main regions of glucose consumption and is notably affected by T2DM. It has been shown that T2DM induces atrophy [15,16,17], fiber-type transition from oxidative to glycolytic [18,19], and impaired energy metabolism in skeletal muscle [20,21,22]. These alterations result in skeletal muscle dysfunction, such as muscle weakness and exercise intolerance [17,23].

It is well known that skeletal muscle is a plastic tissue that easily adapts to changes in energy status due to changes in mitochondria. In healthy individuals, mitochondria are able to switch between carbohydrate and lipid fuels to produce energy in conditions of fasting or nutrients overload. Skeletal muscle of T2DM patients has reduced mitochondrial content [22,24,25]. In patients with insulin resistance, the oxidative capacity of mitochondria is lower than in healthy people, and these changes lead to the accumulation of fat in skeletal tissue [22]. The regulatory signal transmitted to skeletal muscles can be disrupted by changes in the profile of molecules circulating in the plasma, which happens in metabolic disorders [3,4].

In this work, we evaluated the effects of plasma from obese patients and obese patients comorbid with TDM2 on muscle cells functionality. We employed the mouse myoblasts C2C12 differentiation model to test the regulatory effect of patients’ plasma at several levels: (1) cell morphology, (2) functional activity of mitochondria, (3) expression levels of several mitochondria regulators, i.e., *Atgl, Pgc1b*, and miR-378-3p. Mitochondria regulators were selected from the *Atgl*-mediated fat catabolism pathway which was described to control mitochondria function in mouse cardiac muscle and depends on *Atgl*-mediated fatty acid fluxes from lipid droplets [26]. *Atgl* is a rate-limiting enzyme in triacylglycerol hydrolysis, which mediates the production of free fatty acids (FFA) from lipids [27]. FFA represent a substrate for b-oxidation and serve as regulatory ligands for *PPARs* transcription factors [28]. The transcription factor *Pgc1b* is a downstream target of *PPAR*, involved in the coordination of multiple processes in mitochondria, including mitochondrial biogenesis and glucose and fatty acid metabolism [29]. Notably, the first intron of *Pgc1b* encodes miR-378a, which is also known to regulate glucose and fatty acid metabolism and counteract the action of *Pgc1b* on mitochondria activity [30].

## 2. Materials and Methods

### 2.1. Study Population

The study was approved by the Ethics Committee of the Almazov National Medical Research Centre (Ref. # 54/14.03.2016) and was conducted in compliance with current Good Clinical Practice standards and in accordance with the principles under the Declaration of Helsinki (1989). All patients entering the program agreed to and signed an institutional review board-approved statement of informed consent.

### 2.2. Cell Culture and Myogenic Differentiation

The C2C12 mouse myoblasts cell line was purchased from ATCC (ATCC CRL-1772). C2C12 cells were handled as recommended by the manufacturer: cells were cultured in proliferation medium (DMEM supplemented with 4.5 g/L D-glucose, L-glutamine, penicillin–streptomycin, and 20% fetal calf serum (FCS)) and were passaged at 60–80% of confluence. Fusion of some cells without external stimuli usually was observed in sub-confluent cultures and served as a reliable indicator of myogenic commitment, after which we induced differentiation. To induce differentiation, at day 0 the proliferation medium was replaced with differentiation medium (DMEM medium supplemented with 4.5 g/L D-glucose, L-glutamine, penicillin–streptomycin, and 2% horse serum (HS)). The differentiation medium replaced every day; at day 2, after medium replacement, human plasma from healthy volunteers or from patients with obesity and obesity comorbid with diabetes was added to the cultures, as indicated in the corresponding figures’ legends. Cells were harvested, and RNA was extracted for expression analysis on days 0, 2, and 3. Immunocytochemistry was performed on day 4 after stimulation of differentiation.

### 2.3. Plasma Samples Preparation

The blood samples were collected in EDTA tubes, plasma was separated within 2 h after blood collection by two rounds of centrifugation at 1600 *g* for 10 min; supernatant was aliquoted and stored at −80 °C for further processing. For cell culture experiments, samples of plasma were prepared as follows: plasma was thawed, centrifuged at 3000 *g* for 10 min in order to remove the crude pellet; then, centrifugation of the supernatant at 20,000 *g* for 20 min was performed. The obtained supernatant fractions were heated at 55 °C for 30 min to inactivate the complement system and stored at −80 °C until use.

### 2.4. C2C12 Treatment with Plasma

**Figure 1:**
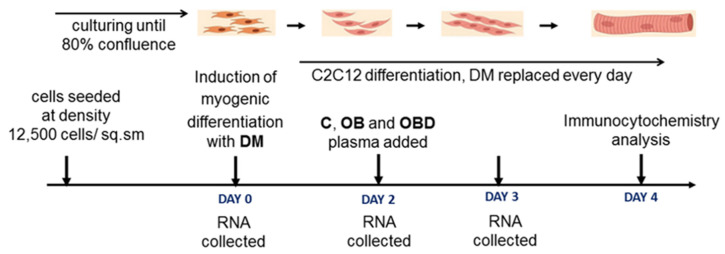
Study design. DM—differentiation medium; plasma from healthy volunteers (C), obese patients (OB), and obese patients comorbid with diabetes (OBD).

The design of the study is summarized in Figure 1. In order to test how cell-free components of blood from patients with obesity and comorbid diabetes affect skeletal muscle development and functions, we compared the effect of plasma from healthy volunteers (C), patients with obesity (OB), or patients with obesity comorbid with diabetes (OBD) on gene expression and morphology of mouse myoblasts C2C12 during myogenic differentiation.

Each sample for cells treatment consisted of pooled plasma from two patients. Plasma was added on day 2 after induction of myogenic differentiation, the time point when the transition between early and late steps of differentiation was observed in our cultures, as described below. RNA samples were collected on days 0, 2, and 3 in order to test the effect of plasma on the dynamics of expression of genes that control mitochondria function and skeletal muscle regeneration and development. In addition, on day 4, differentiated myotubes were fixed and immunostained in order to evaluate myoblast fusion and myotube morphology.

### 2.5. Immunocytochemistry

Cells were grown and differentiated on cover glasses and stained as recommended by the antibody manufacturer. Briefly, cells were washed with PBS buffer, fixed in 4% paraformaldehyde for 10 min at 4 °C, permeabilized with 0.02% Triton X-100 for 5 min. Nonspecific binding was blocked by incubation in 15% FCS for 30 min followed by incubation for one hour with primary anti-myosin heavy chain antibodies (MF20, MAB4470, R&D, USA). Secondary antibodies conjugated with/Alexa-488 (Molecular Probes, Eugene, OR, USA) were applied for 45 min at room temperature. Nuclei were counterstained with DAPI (Molecular Probes, Eugene, OR, USA).

### 2.6. RNA Isolation and Quantification of mRNA of Protein-Coding Genes

Total RNA was isolated using ExtractRNA reagent (Evrogen, cat.no. BC032, Moscow, Russia). cDNA was synthesized from 500 ng of total RNA using a Moloney Murine Leukemia Virus Reverse Transcriptase MMLV RT kit (Evrogen, SK021, Moscow, Russia). A quantitative evaluation of gene expression was performed with qPCR mix-HS SYBR+ROX (Evrogen, cat.no. PK156, Moscow, Russia). Sequences for primers for target amplification are given in Table 1. RT-qPCR data are presented as relative mRNA levels normalized to the geometric mean of the reference transcripts *Gapdh* and *Actb*. All further details are given in corresponding figures’ legends.

### 2.7. MicroRNA Quantification

MicroRNA quantification was performed as described previously [31]. Briefly, for the amplification of microRNAs, TaqMan Universal Master Mix II no UNG and the corresponding TaqMan Assay (Thermo Fisher Scientific, Waltham, MA, USA, ) were used as recommended by the manufacturer. TagMan Assay 001314 was used to detect miR-378a-3p, and Rn00667869_m1 was used to detect to *Actb.* All further details are given in the figures’ legends.

### 2.8. Bioinformatic Analysis and Statististics

TargetScan (http://www.targetscan.org/, accessed on 7 May 2021) [32] and miRDB (http://mirdb.org/, accessed on 7 May 2021) [33] databases were used as sources of computationally predicted targets of microRNA. Enrichment analysis was performed using GO Biological Process pathways gene-set libraries, with ENRICHR software (https://maayanlab.cloud/Enrichr/, accessed on 7 May 2021) [34].

Statistical analysis was carried out using GraphPad Prism 5 software (San Diego, CA, USA). Baseline patients’ characteristics and mean and standard deviation are presented. U-Mann–Whitney test was used to assess differences between the means of two groups. Spearman correlation coefficient was calculated to analyze the relationship between two parameters. *p* values less than 0.01 for enrichment analysis and less than 0.05 for the other experiments were considered statistically significant.

## 3. Results

### 3.1. Description of Patients, Baseline Characteristics

In total, 18 patients were included in this project. All of them were female, 10 recruited patients were obese (BMI > 30 kg/m^2^) (OB group), and 8 had type 2 diabetes mellitus (T2DM) comorbid with obesity (OBD group). Ten healthy female-volunteers with BMI < 18–25 kg/m^2^) were included in the control group (C group). Participants’ clinical characteristics are summarized in Table 2.

Patients with T2DM comorbid with obesity (group OBD) were on metformin treatment monotherapy. Patients with other clinically significant comorbidities and taking drugs that affect body weight (antidepressants, antipsychotics, anorectics) were excluded from the study.

Healthy volunteers were younger (36.7 ± 8.5 years) than patients in the OB and OBD groups (48.0 ± 10.5 years; 52.4 ± 10.4 years, respectively) (*p* < 0.05), while the age of the patients did not differ between the OB and OBD groups. The BMI of patients in the OB and OBD groups did not differ (47.3 ± 8.6 kg/m^2^; 50.4 ± 10.3 kg/m^2^, respectively) and was higher than the BMI of volunteers in the control group (21.8 ± 2.2 kg/m^2^; *p* < 0.001). The HbA1c and glucose levels were significantly higher in OBD patients than in OB patients: 7.5 ± 1.4 % vs. 5.49 ± 0.4 % (*p* < 0.01) and 5.87 ± 0.5 mmol/L vs. 9.0 ± 2.9 mmol/L (*p* < 0.005), respectively. Similar BMI was used as the criterion for plasma pooling, as indicated in the column Pool_ID of Table 2.

### 3.2. Plasma from Obese Patients (OB) and Patients with Type 2 Diabetes Comorbid with Obesity (OBD) Affects the Expression of Mymk and the Composition of Myofiber Types during Skeletal Muscle Differentiation

**Figure 2:**
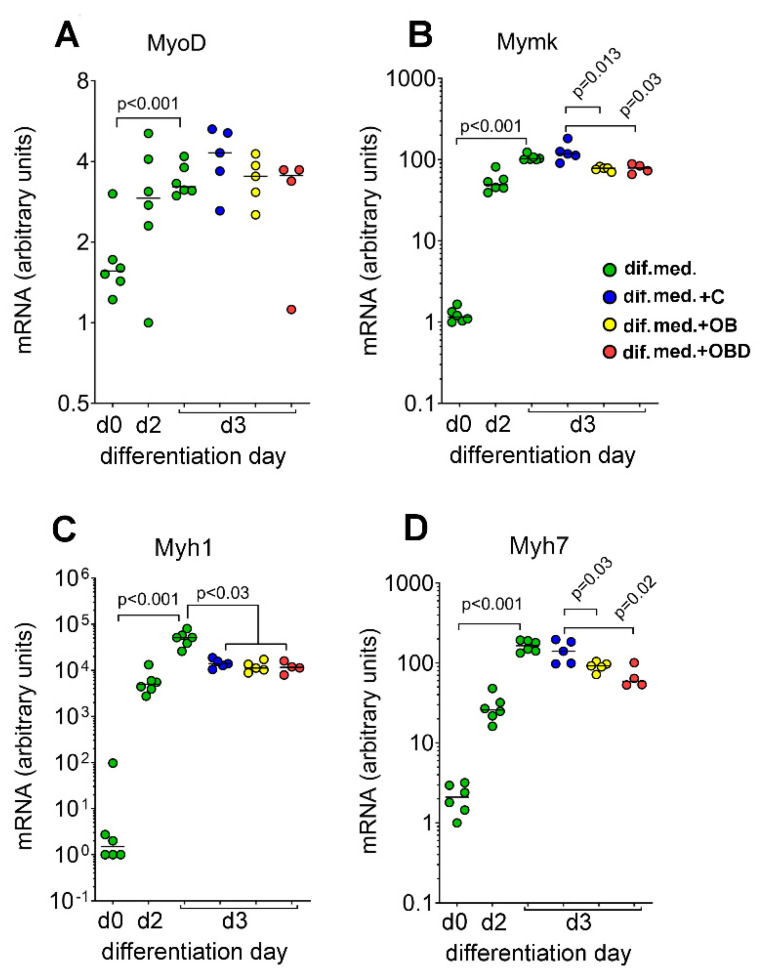
Effects of plasma from obese patients (OB), patients with type 2 diabetes comorbid with obesity (OBD), and healthy volunteers (C) on the expression of markers of myogenic differentiation in C2C12 myoblasts. (**A**) Myogenic regulatory factor *MyoD*; (**B**) muscle-specific myoblast fusion regulator *Mymk*; (**C**) fast-glycolytic fiber *Myh1* isoform; (**D**) oxidative-slow fiber *Myh7* isoform.

First, we tested how plasma from obese patients (OB) and patients with type 2 diabetes comorbid with obesity (OBD) affect skeletal muscle growth and development using the C2C12 mouse myoblasts differentiation model. In order to choose the best time point to treat the differentiating C2C12 myoblasts, we determined when transition from early to late stage of differentiation occurs. In C2C12 cultures treated with differentiation medium, there is a well-coordinated pattern of expression of the genes *MyoD* [35] and *Mymk*, [36] which regulate early events of myogenesis, and of those for myosins *Myh1/Myh7*, which indicate the successful transition to the myofiber maturation stage of myogenesis [35]. Here, we show that expression of the myogenic regulatory factor gene *MyoD* and of the gene *Mymk* that regulates myoblast fusion strongly increased during transition from day 0 to day 2 and then either remined at the same level (*MyoD*) or showed a small but significant increase by day 3 (*Mymk*) (Figure 2A,B; green label). On the contrary, the expression of the fast-twitch fiber marker *Myh1* and of the slow-twitch fiber marker *Myh7* showed a stable increase during transition from day 0 to day 3 (Figure 2C,D; green label). Together, these data show that the transition between the early steps of differentiation to fiber growth and maturation occurred in our C2C12 myoblasts between day 2 and day 3 after stimulation; therefore, we chose day 2 as the most appropriate time for plasma treatment.

The analysis of the expression of genes that control myogenesis during the transition from day 2 to day 3 of myogenic differentiation revealed substantial alterations: while the expression of *MyoD* did not differ in cells subjected to different treatments (DifMed/C/OB/OBD, Figure 2A), the expression of *Mymk* was downregulated by OB/OBD plasma, but not by plasma from healthy volunteers (C) (Figure 2B). Furthermore, in all plasma-treated samples, we detected downregulated expression of fast-twitch glycolytic *Myh1*, while the expression of slow-twitch oxidative *Myh7* was downregulated in cultures treated with OB/OBD plasma in comparison to DifMed/C cultures (Figure 2C,D).

**Figure 3:**
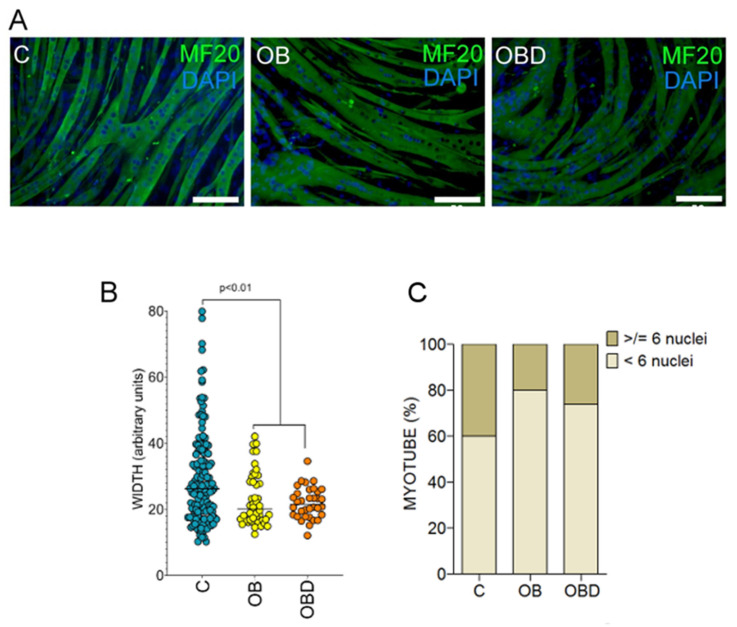
Myotubes morphology and fusion coefficient. (**A**) Immunocytological staining of differentiated myotubes with anti-myosin heavy chain antibody MF20 in C2C12 cultures treated with plasma from C, OB, and OBD patients on day 5 after stimulation. Scale bars represent 100 μm; (**B**) width of myotubes in C/OB/OBD-treated cultures, measured in arbitrary units in 5–6 independent photographs; the number of myotubes on each photo was 10–20; (**C**) proportion of myotubes containing ≥6 nuclei or <6 nuclei in C/OB/OBD-treated cultures (%).

The differences in morphology of C2C12 myotubes treated with C/OB/OBD plasma confirmed the alterations in myoblasts fusion activity in OB/OBD-treated cultures: the width of OB/OBD-treated myotubes was significantly smaller than that of C-treated myotubes, and the fraction of tubules with a high number of nuclei was larger in C-treated myotubes (Figure 3A–C).

### 3.3. Plasma from Obese Patients (OB) and from OB Patients Comorbid with Type 2 Diabetes (OBD) Affects C2C12 Myotubes Bioenergetics

**Figure 4:**
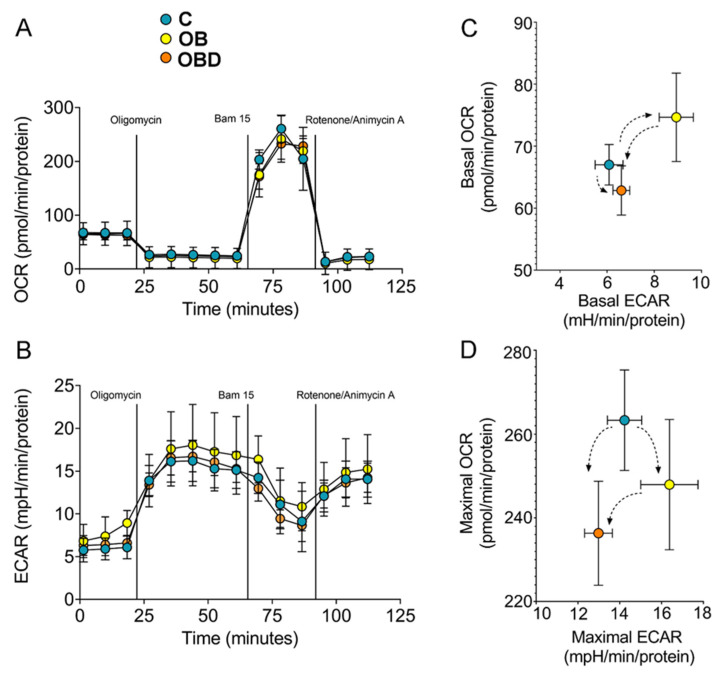
Metabolic profiles of C2C12 myotubes treated with C, OB, and OBD plasma. (**A**) OCR traces for myotubes treated with human plasma, as indicated; (**B**) kinetic profiles of ECAR for myotubes treated with human plasma, as indicated; (**C**) basal OCR vs. basal ECAR [mean ± sem for both parameters]; (**D**) Maximal Respiratory capacity vs. Maximal Glycolytic capacity [mean ±  sem]. All data were calculated from 6–8 Seahorse microplate wells and were normalized to total protein content in each well.

To investigate the effect of plasma from OB/OBD patients on cellular bioenergetics, a mitochondria stress test was performed on C/OB/OBD-treated myotubes. This technique allows quantifying OXPHOS and glycolysis by using a specific protocol designed to target distinct components of these pathways with pharmacological agents. Basal measurements of the mitochondrial respiration rate (OCR) and glycolytic ECAR were performed in C2C12 myotubes treated with C/OB/OBD plasma (Figure 4A,B). The OCR vs. ECAR plot showed increased ECAR in OB-treated myotubes but not in OBD-treated myotubes compared to myotubes treated with plasma from C participants, suggesting a metabolic profile switch to glycolysis in OB- but not in OBD-treated myotubes (Figure 4C). The mitochondrial uncoupler BAM15 was used to estimate the maximal respiration rate, and myotubes treated with OBD but not with OB plasma showed a significant decrease in the maximal respiration rate (Figure 4D).

These findings indicate that plasma from OB and OBD patients affected the bioenergetics of C2C12 myotubes in different ways: while OBD plasma negatively affected the maximal mitochondrial respiration rate, OB plasma stimulated the metabolic switch to glycolysis.

### 3.4. Plasma from Obese Patients (OB) and Patients with Type 2 Diabetes Comorbid with Obesity (OBD) Affects the Expression of the Mitochondria Regulators Atgl/Pgc1b/miR378-a-3p

Then, we tested how plasma from obese patients with or without comorbid type 2 diabetes affected the expression of mitochondria regulators using the C2C12 mouse myoblasts differentiation model.

**Figure 5:**
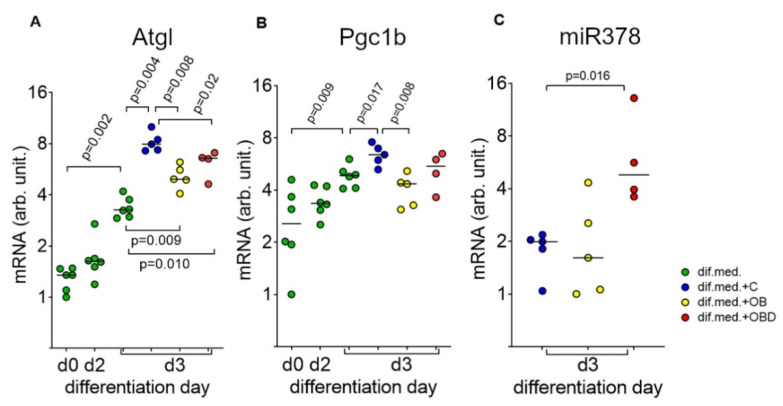
Effects of plasma from healthy volunteers (C), obese patients (OB), and obese patients comorbid with type 2 diabetes (OBD) on ethe xpression of mitochondria regulators in C2C12 myoblasts. (**A**) Adipose triglyceride lipase (*Atgl*); (**B**) *PPARG* co-activator (*Pgc1b*); (**C**) miR-378a (miR378a).

**Figure 6:**
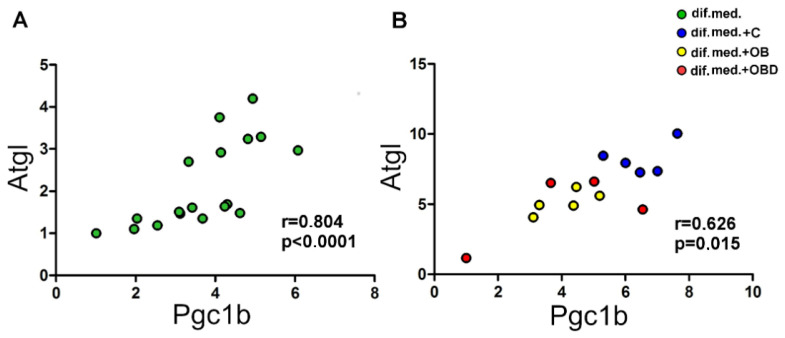
Correlation between the levels of *Atgl* and those of *Pgc1b* (**A**) during C2C12 differentiation and (**B**) after treatment of C2C12 with plasma.

During C2C12 differentiation from day 0 to day 3, the levels of both adipose triglyceride lipase (*Atgl*) and *PPARG* coactivator (*Pgc1b*) increased up to 2.4-fold and 1.9-fold, respectively (Figure 5A,B; green label). The plasma of obese patients had a stronger effect on the levels of both *Atgl* and *Pgc1b* than the plasma of obese patients with type 2 diabetes. Specifically, the levels of *Atgl* were downregulated 1.6-fold by plasma from OB patients and 1.2-fold by plasma from OBD patients compared to plasma from healthy volunteers (Figure 5A; blue, yellow, and red labels). The levels of *Pgc1b* was downregulated 1.5-fold by plasma from OB patients and remained unchanged after treatment with plasma from OBD patients compared to plasma from healthy volunteers (Figure 5B; blue, yellow, and red labels). At the same time, changes in the levels of miR-378a-3p in response to plasma treatment were different—the levels remained unchanged after treatment with plasma from OB patients and were upregulated 2.4-fold by plasma from OBD patients compared to plasma from healthy volunteers (Figure 5C; blue, yellow, and red labels). Positive correlation was observed between levels of *Atgl* and *Pgc1b* both during C2C12 differentiation (Figure 6A) and after plasma treatment (Figure 6B).

### 3.5. Analysis of Existing Databases of mir-378a-3p Potential Targets to Predict Downstream Cellular Processes

To explore the potential relationship between changes in the level of miR-378a-3p expression and specific cellular processes in skeletal muscle tissue, we analyzed existing databases to characterize the regulatory potential of miR-378-3p. Predicted protein targets of miR-378a-3p were retrieved from TargetScan and miRDB databases. Overlapping targets in these sets, which contained 69 genes, were used for enrichment analysis. The GO Biological Processes associated with predicted targets of miR-378a-3p are presented in Table 3. Notably, among the identified GO Biological Processes, several pathways were related directly to skeletal muscle development, such as “regulation of muscle hypertrophy” and “myoblast differentiation”; the others pathways were related to general metabolic processes, such as “regulation of macromolecule metabolic process”, “MAPK cascade”, “cellular response to glucocorticoid stimulus”.

## 4. Discussion

In this work, we showed that plasma from OB and OBD patients affects skeletal muscle growth, development, and metabolism. The most important observations made in this study are that OB/OBD plasma affects skeletal muscle mitochondrial function.

We showed that treatment of C2C12 with OB samples caused a decrease in the expression of the *Atgl* and *Pgc1b* genes, which suggested that the plasma of these patients contains regulatory molecules that suppress the expression of these genes. At the same time, the energetics of the cells shifted towards glycolysis, probably due to a decrease in the content of fatty acids as a substrate, and the respiration rate of mitochondria was higher than in other samples. Decreased expression of the *Pgc1b* gene may also affect the morphology of myotubes, since *Pgc1a* and *Pgc1b* are known to attenuate protein synthesis and degradation in skeletal muscle through mechanisms dependent on estrogen-related receptor alpha (ERRα) [37].

An increased level of miR-378-3p was found in myotubes treated with OBD plasma. There is evidence that miR-378-3p is involved in mitochondrial energy homeostasis, inhibiting mitochondrial function, and affects the development, differentiation, and regeneration of muscles [38,39,40]. Bioenergetic analysis using SeaHorse demonstrated that OBD plasma inhibited mitochondrial respiration. This can also be explained by the fact that all OBD patients were on metformin therapy, which impairs mitochondrial respiration. According to the literature, metformin reduces the efficiency of mitochondrial metabolism, leading to a switch to aerobic glycolysis [41,42,43]. However, in our work, in the samples treated with OBD plasma, there was no shift towards glycolysis; it is probably more difficult for diabetics to shift the balance in this direction. Only changes in the expression of the *Atgl* gene were observed, as observed also in the samples treated with OB plasma (there was no additional effect of diabetes compared to obesity).

The correlation between *Atgl* and *Pgc1b* levels both during C2C12 differentiation and after plasma treatment suggests that these genes form a regulatory unit in different states of C2C12 cells. The absence of a positive correlation between the levels of *Pgc1b* and those of its encoded intron miR-378-3p confirms the recent discovery that miR-378 has its own transcription apparatus [44].

We believe that the system used in this work (model of skeletal muscle cells + short-term exposure to readily available plasma samples of patients) may be useful for predicting the state of skeletal muscle in patients with metabolic disorders.

Indeed, the changes in mitochondrial functions that we observed in C2C12 are consistent with known changes in patients’ skeletal muscles, namely, stronger impairment of skeletal muscle mitochondrial function in obese patients with diabetes compared to obese patients without diabetes [45,46,47].

It is noteworthy that all the studied targets are involved in the regulation of fatty acid oxidation: *Atgl* provides a controlled release of fatty acids [27], *Pgc1b* acts as an activator of fatty acid oxidation [48], and miR-378-3p acts as an inhibitor [30]. The observed changes in the levels of each target have a negative regulatory effect on fatty acid oxidation, which is consistent with the fact that mitochondrial fatty acid oxidation is reduced in skeletal muscle of diabetic patients [49,50]. The accumulation of lipids in skeletal muscle decreases their insulin sensitivity [51].

The effects of *Atgl* on mitochondria functions seem to be tissue-specific. In the heart, FFA produced by *Atgl* is involved in *PPARa* activation, which regulates mitochondrial FFA oxidation and prevents lipid accumulation in the myocardium [26], whereas in skeletal muscle, no alteration in mitochondrial content or respiration functions were found after manipulation of *Atgl* levels in mouse models [52,53]. On the other hand, the use of the C2C12 skeletal muscle cell model allowed to reveal a link between *Atgl* and mitochondrial functions (*Atgl* overexpression was shown to increase lipolysis from intramyocellular lipid droplets, activate transcriptional responses (*PPARd* and its target *Pgc1a*) to enhance mitochondrial fatty acid oxidative capacity, and protect against the deleterious effect of high fat levels on mitochondria function), probably due to the lack of overlap with other metabolic pathways which are active in animal models [54,55].

**Figure 7:**
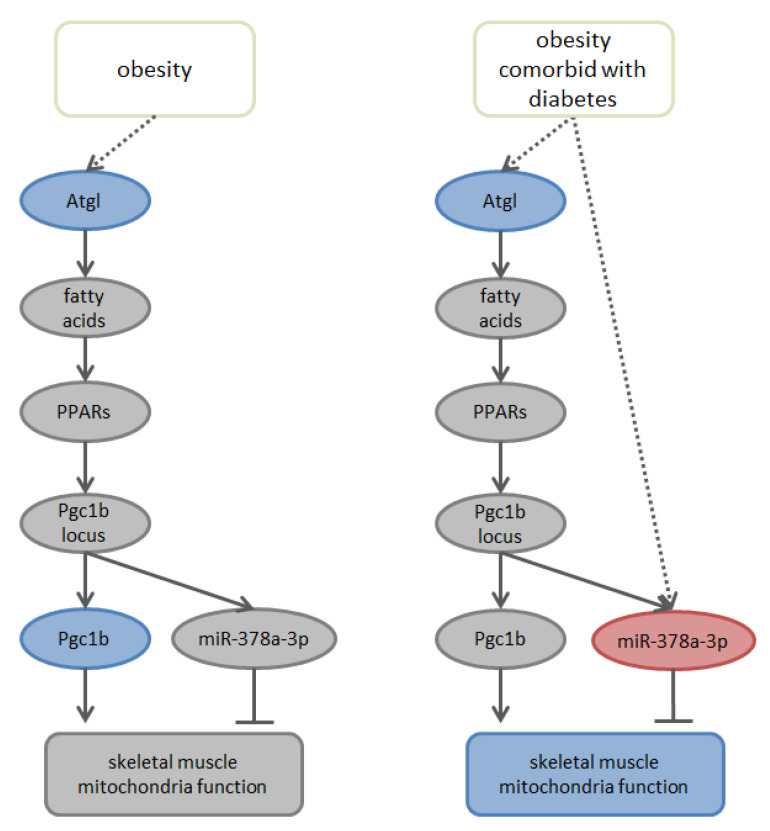
Proposed model of patients’ plasma effects on mitochondria function and on the levels of its regulators. (Blue and red—observed down- and up-regulation).

Two types of the changes in gene expression were identified: (i) one type, common to plasma from both groups of patients, was a decrease in *Atgl* levels, and (ii) the other type, specific for plasma from obese and diabetic patients, was an increase in miR-378-3p levels. Figure 7 shows a suggested model of the possible effect of patient plasma on mitochondrial function and levels of its regulators, which summarizes our results and literature data. Earlier, it was shown that *Atgl* has an indirect positive effect on mitochondrial functions [56], while miR-378-3p negatively regulates the function of mitochondria [30]. Therefore, it can be assumed that when C2C12 are exposed to plasma of obese and diabetic patients, both identified components combine, and their synergistic action causes a stronger dysfunction of mitochondria.

Enrichment analysis data suggest that miR-378-3p may also interfere with MAPK signaling pathway through MAPK1 (p38 Map kinase), known to be involved in the regulation of myogenesis, as suggested by the link between ROS, accumulating in proliferating satellite cells, and activation of p38α MAP kinase leading to satellite cell differentiation [56]. The dysregulation of p38 Map kinase signaling contributes to skeletal muscle wasting, sarcopenia, and skeletal muscle stem cells premature senescence [57]. These mechanisms might provide the link between miR-378a-3p, mitochondrial dysfunction, and functional metabolic alterations in skeletal muscle in obese patients with TDM2. Further investigations are required to clarify this issue.

In conclusion, we demonstrated here that, even during short-term exposure, plasma from patients with obesity and obesity comorbid with diabetes possess disease-specific regulatory action towards differentiation characteristics and bioenergetics properties of C2C12 myoblasts.

The study has potential limitations that could be addressed in future research. First, difference in age between the control and OB or OBD groups can introduce a bias, when analyzing the data between control and OB or OBD groups. Second, some small differences between groups may be missed due to small number of enrolled patients. Third, the present investigation lack data on such metabolic parameters as triglycerides and HDL-cholesterol.

## Figures and Tables

**Table 1:** Sequences for primers.

Target	Sequence 5′–3′
*Myh1 forward*	GCTGAGAGAAGCTACCACATT
*Myh1 reverse*	ACAAAGGCGTAGTCGTATGG
*Myh7 forward*	TGCCCGATGACAAAGAAGAG
*Myh7 reverse*	GTCACCGTCTTGCCATTCT
*Mymk forward*	CCTGTGATGGGCCTGGTTTGTC
*Mymk reverse*	GGTTCATCAAAGTCGGCCAGTGC
*MyoD forward*	TGCCTTCTACGCACCTGGA
*MyoD reverse*	CGCTGTAATCCATCATGCCATC
*Atgl forward*	CCACTTTAGCTCCAAGGATGAG
*Atgl reverse*	TTGGAGGGTAGGAGGAATGA
*Pgc1b forward*	CTTTATCTCTTCCTCTGACCCCAG
*Pgc1b reverse*	CCAGAGAGTTCCACACTTAAGGTT
*Gapdh forward*	GGATCTGACGTGCCGCCTG
*Gapdh reverse*	GAAGGTGGA AGAGTGGGAGTTGC

**Table 2:** Patients’ characteristics.

Group of Patients	Patiet ID	Age (Years)	BMI (kg/m^2^)	Obesity Grade	Diabetes Duration (Years)	HbA1c Levels (%)	Glucose Levels (mmol/L)	Pool_ID	Pool_BMI
Control (C)	c1	26	16.9	NA	NA	5.2	5.06	C1	18.7
	c2	26	20.6	NA	NA	4.5	4.65	C1	18.7
	c8	43	20.7	NA	NA	5.5	5.24	C2	21.0
	c11	38	21.3	NA	NA	4.7	4.9	C2	21.0
	c9	45	22.2	NA	NA	5.1	5.0	C3	22.2
	c4	50	22.2	NA	NA	5.1	5.38	C3	22.2
	c10	32	22.4	NA	NA	5.0	5.38	C4	22.9
	c5	44	23.4	NA	NA	5.6	5.24	C4	22.9
	c7	33	24.1	NA	NA	4.5	4.39	C5	24.2
	c3	30	24.4	NA	NA	4.5	4.39	C5	24.2
Obesity (OB)	B122	40	40.2	III	NA	5.75	6.29	OB1	40.6
	B136	35	40.9	III	NA	5.14	6.18	OB1	40.6
	B130	57	41.2	III	NA	5.4	5.06	OB2	41.7
	B131	61	42.2	III	NA	5.7	5.9	OB2	41.7
	B141	40	43.9	III	NA	5.6	5.8	OB3	44.4
	B164	53	44.8	III	NA	5.5	5.63	OB3	44.4
	B160	34	46	III	NA	5.0	5.18	OB4	48.2
	B151	60	50.4	III	NA	5.3	5.99	OB4	48.2
	B147	56	56.4	III	NA	5.2	5.8	OB5	61.9
	B124	44	67.3	III	NA	6.4	6.84	OB5	61.9
Obesity comorbid with diabetes (OBD)	B158	60	36.2	II	8	6.2	8.77	OBD1	37.6
	B202	61	38.9	II	17	6.8	9.07	OBD1	37.6
	B174	46	47.2	III	13	6.8	6.63	OBD2	48.1
	B169	66	48.9	III	7	7.09	7.19	OBD2	48.1
	B168	56	49.8	III	3	7.87	8.35	OBD3	51.8
	B128	39	53.8	III	5	9.2	12.58	OBD3	51.8
	B112	53	63.2	III	7	6.56	5.25	OBD4	64.2
	B142	38	65.1	III	7	10.05	14.49	OBD4	64.2

Obesity grade II (35.0 ≤ BMI ≤ 39.9 kg/m^2^) and obesity grade III (BMI ≥ 40.0 kg/m^2^).

**Table 3:** GO Biological Processes associated with predicted targets of miR-378a-3p.

GO Term	*p*-Value	Predicted Targets of miR-378a-3p
ERK1 and ERK2 cascade (GO:0070371)	0.00005	MAPK1; IGF1; ZFP36L2
negative regulation of stem cell differentiation (GO:2000737)	0.00052	REST; ZFP36L2
nervous system development (GO:0007399)	0.00096	NEUROD1; DSCAM; PAX8; DYRK1A; DCX; SHANK3; DACT1
regulation of macromolecule metabolic process (GO:0060255)	0.00103	MAPK1; ALPK3; IGF1; TOB2
positive regulation of muscle hypertrophy (GO:0014742)	0.00104	IGF1; IL6ST
cellular response to corticosteroid stimulus (GO:0071384)	0.00120	REST; ZFP36L2
synapse assembly (GO:0007416)	0.00135	NPAS4; DSCAM; SHANK3
positive regulation of cardiac muscle hypertrophy (GO:0010613)	0.00136	IGF1; IL6ST
myoblast differentiation (GO:0045445)	0.00136	REST; IGF1
cellular response to glucocorticoid stimulus (GO:0071385)	0.00154	REST; ZFP36L2
peptidyl-serine phosphorylation (GO:0018105)	0.00159	DYRK1A; MAPK1; CSNK1G2; CAMKK2
peptidyl-threonine phosphorylation (GO:0018107)	0.00168	DYRK1A; MAPK1; CAMKK2
positive regulation of transcription regulatory region DNA binding (GO:2000679)	0.00193	NEUROD1; IGF1
phosphorylation (GO:0016310)	0.00214	TOLLIP; DYRK1A; MAPK1; ALPK3; CSNK1G2; CAMKK2
regulation of cardiac muscle hypertrophy (GO:0010611)	0.00236	IGF1; IL6ST
MAPK cascade (GO:0000165)	0.00272	MAPK1; IGF1; SHANK3; ZFP36L2; CAMKK2
peptidyl-serine modification (GO:0018209)	0.00283	DYRK1A; MAPK1; CSNK1G2; CAMKK2
modulation of chemical synaptic transmission (GO:0050804)	0.00286	NPAS4; RIMS4; GRIK3
response to glucocorticoid (GO:0051384)	0.00308	REST; ZFP36L2
peptidyl-threonine modification (GO:0018210)	0.00361	DYRK1A; MAPK1; CAMKK2
regulation of transcription regulatory region DNA binding (GO:2000677)	0.00361	NEUROD1; IGF1
response to hexose (GO:0009746)	0.00389	NEUROD1; IGF1
positive regulation of cell differentiation (GO:0045597)	0.00454	NEUROD1; PAX8; IGF1; IL6ST
positive regulation of gene expression (GO:0010628)	0.00494	NEUROD1; REST; PAX8; DYRK1A; MAPK1; IGF1; RBMS3; CAMKK2
negative regulation of fat cell differentiation (GO:0045599)	0.00544	ZFPM2; ZFP36L2
positive regulation of signal transduction (GO:0009967)	0.00561	TSPAN17; IGF1; SHANK3; DACT1
protein phosphorylation (GO:0006468)	0.00561	DYRK1A; MAPK1; ALPK3; CSNK1G2; CAMKK2; MAP2K6
regulation of interferon-beta production (GO:0032648)	0.00613	YY1; TRAF3
positive regulation of DNA binding (GO:0043388)	0.00613	NEUROD1; IGF1
positive regulation of osteoblast differentiation (GO:0045669)	0.00648	IGF1; IL6ST
epithelium development (GO:0060429)	0.00737	PAX8; TOLLIP; DACT1
sphingolipid metabolic process (GO:0006665)	0.00754	SERINC1; KDSR; CSNK1G2
activation of MAPK activity (GO:0000187)	0.00772	MAPK1; IGF1; MAP2K6
regulation of gene expression (GO:0010468)	0.00905	YY1; REST; ZNF514; MAPK1; ALPK3; IGF1; TOB2; SRSF10; RBMS3
negative regulation of macromolecule metabolic process (GO:0010605)	0.00905	YY1; REST; RBMS3

## Data Availability

Publicly available datasets were analyzed in this study. This data can be found here: [http://www.targetscan.org/, accessed on 20 May 2021; http://mirdb.org/, accessed on 20 May 2021]. The data presented in this study are available on request from the corresponding author. The data are not publicly available due to privacy restrictions.
